# Evidence of Notch-Hesr-Nrf2 Axis in Muscle Stem Cells, but Absence of Nrf2 Has No Effect on Their Quiescent and Undifferentiated State

**DOI:** 10.1371/journal.pone.0138517

**Published:** 2015-09-29

**Authors:** Masahiko Yamaguchi, Satoshi Murakami, Tomohiro Yoneda, Miki Nakamura, Lidan Zhang, Akiyoshi Uezumi, Sumiaki Fukuda, Hiroki Kokubo, Kazutake Tsujikawa, So-ichiro Fukada

**Affiliations:** 1 Laboratory of Molecular and Cellular Physiology, Graduate School of Pharmaceutical Sciences, Osaka University, 1-6 Yamadaoka, Suita, Osaka 565-0871, Japan; 2 Division for Therapies Against Intractable Diseases, Institute for Comprehensive Medical Science, Fujita Health University, 1-98 Dengakugakubo, Kutsukake, Toyoake, Aichi 470-1192, Japan; 3 Department of Cardiovascular Physiology and Medicine, Graduate School of Biomedical and Health Sciences, Hiroshima University, 1-2-3 Kasumi, Minamiku, Hiroshima 734-8551, Japan; University of Rome La Sapienza, ITALY

## Abstract

Nrf2 is a master regulator of oxidative stresses through the induction of anti-oxidative genes. Nrf2 plays roles in maintaining murine hematopoietic stem cells and fly intestinal stem cells. The canonical Notch signaling pathway is also crucial for maintaining several types of adult stem cells including muscle stem cells (satellite cells). Here, we show that Dll1 induced Nrf2 expression in myogenic cells. In addition, primary targets of Notch signaling, Hesr1 and Hesr3, were involved in the up-regulation of Nrf2 mRNA and expression of its target genes. *In vitro*, Nrf2 had anti-myogenic and anti-proliferative effects on primary myoblasts. *In vivo*, although Nrf2-knockout mice showed decreased expression of its target genes in muscle stem cells, adult muscle stem cells of Nrf2-knockout mice did not exhibit the phenotype. Taken together, in muscle stem cells, the Notch-Hesr-Nrf2 axis is a pathway potentially inducing anti-oxidative genes, but muscle stem cells either do not require Nrf2-mediated anti-oxidative gene expression or they have a complementary system compensating for the loss of Nrf2.

## Introduction

Canonical Notch signaling is essential for maintaining several types of adult stem cells including muscle stem cells, melanocyte stem cells, neural stem cells, epithelial stem cells, and intestinal stem cells [1–4]. When Notch is activated, its intracellular domain is cleaved by γ-secretase and it translocates to the nucleus, where it activates the transcription of target genes through interaction with Rbpj (Recombination Signal-Binding Protein 1 for J-Kappa) and MamL (mastermind-like). Well-known targets of canonical Notch signaling are *Hes* (Hairy and enhancer of split) and *Hesr* (Hes-related, also known as *Hey/Herp/Hrt/Gridlock/Chf*) family genes [5, 6]. Using genetic tools to conditionally abrogate *Rbpj* in muscle stem cells, two independent groups reported that canonical Notch signaling was essential for maintaining muscle stem cells in a quiescent and undifferentiated state in mouse adult skeletal muscle [3, 4]. We and other groups previously reported that, in myogenic cells, Hesr1 and Hesr3 are predominantly induced by Notch ligand (Dll1 or Dll4) [7], and that Hesr1 and Hesr3 are essential for the generation of adult muscle stem cells during postnatal development [8]. Like *Rbpj*-knockout muscle stem cells, *Hesr1/Hesr3*-double knockout (dKO) muscle stem cells express myogenic and proliferation-related proteins that are rarely detected in adult muscle stem cells. Therefore, the Notch/Rbpj/Hesr1-Hesr3 pathway is considered to be essential for generating adult muscle stem cells, but the molecular mechanism by which quiescent and undifferentiated states are impaired in the absence of Hesr1 and Hesr3 is still unknown [9].

Nrf2 is ubiquitously expressed in cells and is well characterized as a master regulator of the anti-oxidative response pathway. Nrf2 activity is tightly regulated by Keap1 (Kelch-like erythroid cell-derived protein with CNC homology [ECH]-associated proteins). Intriguingly, Hochmuth et al. demonstrated that CncC (a homologue of Nrf2) activity is related to aged-related loss of intestinal stem cells in *Drosophila* [10]. CncC induces antioxidant genes, which result in low oxidative stress and maintain intestinal stem cells in the quiescent state. However, Keap1 suppressed the transcriptional activities of CncC in old flies and led to decreased expression of antioxidant genes, which resulted in a high ROS (Reactive oxygen species) and proliferative condition leading to aged-related degeneration of the intestinal epithelium. Tsai et al. reported the importance of Nrf2 in murine hematopoietic stem cells. In *Nrf2*-knockout mice, hematopoietic stem cell and progenitor pools are expanded, which indicates an intrinsic dysfunction of hematopoietic stem cells in their migration to and retention in the niche [11]. However, the role of Nrf2 in muscle stem cells remains to be elucidated. In addition, information about the transcriptional regulation of Nrf2 is limited compared to that of its post-transcriptional regulation.

Intriguingly, the Notch1 promoter has an Nrf2-binding element (ARE), and Nrf2 directly regulates Notch expression [12]. In addition, the same group recently demonstrated that the Nrf2 promoter has an Rbpj binding element and that canonical Notch signaling directly regulates Nrf2 gene expression [13]. Thus, these two major signaling molecules may reciprocally regulate each other, but, in adult stem cells, the relationship between Nrf2 and Notch signaling is still largely unknown.

Here, we show the presence of a Notch-Nrf2 axis in muscle stem cells. We also found regulation of Nrf2 expression by the primary target of Notch signaling, Hesr1 and Hesr3. Similar to Notch signaling, Nrf2 has an anti-myogenic effect on myogenic cells *in vitro*. In addition, the loss of Nrf2 resulted in decreased expression of its target genes in *in vivo* muscle stem cells. However, *Nrf2*-knockout mice did not exhibit any abnormality in muscle stem cells. These results indicate that the Notch-Hesrs-Nrf2 axis is a potential active pathway in muscle stem cells, but that Nrf2 is dispensable, or it has redundant genes or a redundant pathway in muscle stem cells.

## Results

### Notch signaling induced Nrf2 mRNA in myogenic cells

**Fig 1:**
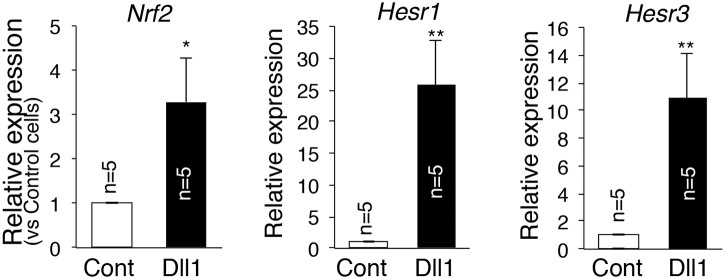
Dll1 induces mRNA expression of *Nrf2*. C2C12 cells were cultured with CHO (white) or CHO-Dll1 (black). After 12 hours co-culture, total RNA was obtained and *Nrf2* and Notch effector gene expressions were examined by real-time PCR. The *y*-axis shows the mean±S.D. (n = 5). **, *P*<0.01, *, *P*<0.05.

First, we examined the induction of Nrf2 mRNA by Notch ligand in myogenic cells. C2C12 cells, a myogenic cell line, were cultured with Dll1-expressing CHO (Chinese Hamster Ovary) cells (Dll1) or control CHO cells (Cont), and then we compared mRNA expressions of Nrf2 by Dll1 and Cont cultures. Similar to Hesr1 and Hes3 (well-known primary targets of Notch signaling), Nrf2 expression was upregulated in C2C12 cells co-cultured with Dll1 (Fig 1). These results indicate the presence of a Notch-Nrf2 axis in myogenic cells.

**Fig 2:**
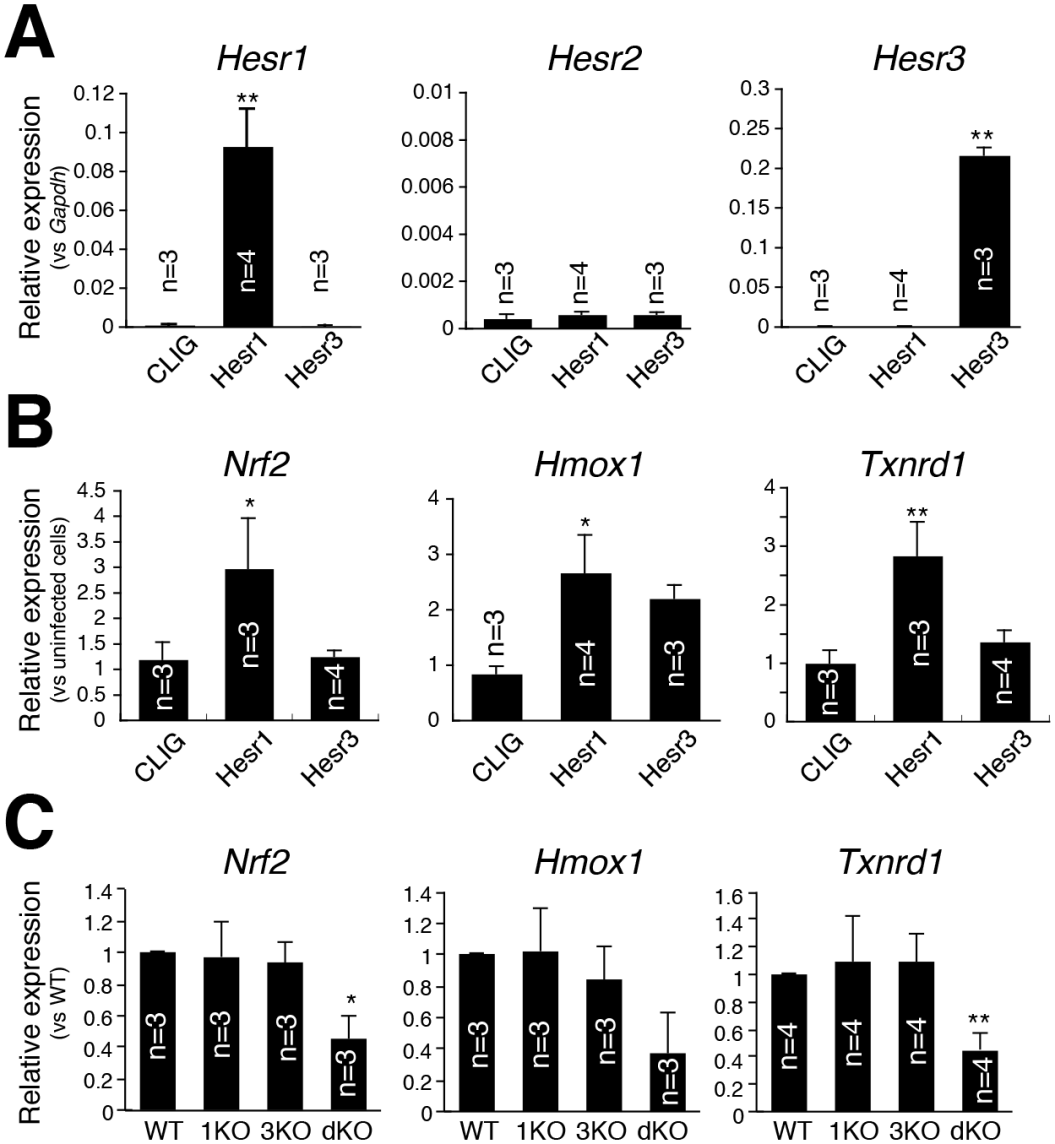
Hesr1 and Hesr3 affect *Nrf2* expression. (A) Relative expressions of *Hesr1*, *Hesr2*, and *Hesr3* mRNA were compared among control (CLIG), Hesr1-overexpressed (Hesr1), and Hesr3-overexpressed (Hesr3) primary myoblasts. The *y*-axis indicates means±S.D. (n = 3–4). **, *P*<0.01. (B) Relative expressions of *Nrf2*, *Hmox1*, and *Txnrd1* mRNA were compared among control (CLIG), Hesr1-overexpressed (Hesr1), and Hesr3-overexpressed (Hesr3) primary myoblasts. The *y*-axis indicates means±S.D. (n = 3–4). **, *P*<0.01, *, *P*<0.05. (C) Relative expressions of *Nrf2*, *Hmox1*, and *Txnrd1* mRNA were compared among wild-type (WT), *Hesr1*-null (1KO), *Hesr3*-null (3KO), and *Hesr1*/*Hesr3*-double null (dKO) MuSCs. The *y*-axis indicates means±S.D. (n = 3–4). **, *P*<0.01, *, *P*<0.05.

Next, we examined whether Hesr1 and Hesr3 induced the mRNA expressions of Nrf2 and its target genes, Hmox1 and Txnrd1. Hesr1 or Hesr3 was retrovirally transduced in primary myoblasts (Fig 2A), and we observed that Hesr1 significantly induced Nrf2 and its target genes, Hmox1 (Heme Oxygenase-1) and Txnrd1 (Thioredoxin reductase 1) (Fig 2B). We also investigated the mRNA expression of Nrf2 in *Hesr1*- and/or *Hesr3*-knockout MuSCs. In our previous analyses, MuSCs in *Hesr1*- or *Hesr3*-single knockout mice did not show a remarkable phenotype [8]. Consistent with previous results, *Hesr1*- or *Hesr3*-single knockout MuSCs expressed a level of Nrf2 similar to that of wild-type mice. However, *Hesr1*/*Hesr3*-double knockout MuSCs exhibited decreased expressions of Nrf2 and its target genes (Fig 2C). These results suggest that Hesr1 and Hesr3 are necessary for Nrf2 expression in MuSCs.

**Fig 3:**
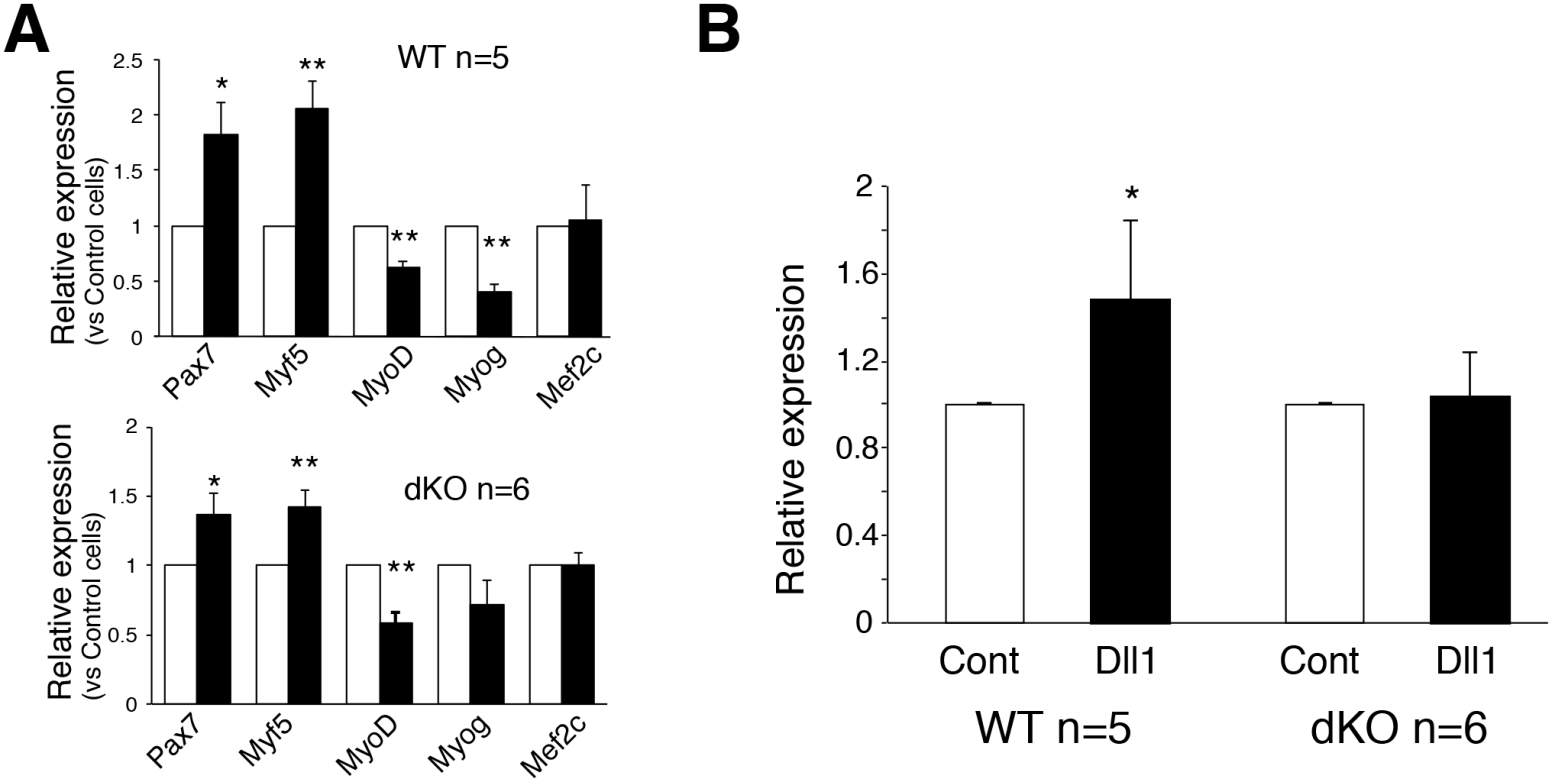
*Nrf2* expression in *Hesr1*/*Hesr3*-double null primary myoblasts is decreased. (A) Relative expressions of *Pax7*, *Myf5*, *MyoD*, and *Myogenin* mRNA by myoblasts co-cultured cells with CHO (white) and CHO-Dll1 were compared (black). The *y*-axis indicates means±S.D. (n = 5–6). (B) Primary myoblasts derived from control (WT) or *Hesr1*/*Hesr3*-double null (dKO) were cultured with CHO (Cont) or CHO-Dll1 (Dll1). After 24 hours co-culture, total RNA was obtained and myogenic genes and *Nrf2* gene expression examined by real-time PCR. (n = 5–6).

In order to examine the contributions of Hesr1 and Hesr3 to Dll1-dependent Nrf2 expression in primary myoblasts, similar to Fig 1, primary myoblasts were co-cultured with Dll1-expressing CHO or control CHO cells. In control cells, Nrf2 was induced by Dll1 as well as Pax7 (paired box 7) and Myf5 (myogenic factor 5) (Fig 3A and 3B). In contrast, the expressions of MyoD (myogenic determination gene) and myogenin were decreased by Dll1. This increment or decrement was also observed in dKO myoblasts. However, the increased expression of Nrf2 was not observed in dKO myoblasts. Taken together, these results indicate that canonical Notch pathways induced mRNA expression of Nrf2 potentially in a Hesr1/Hesr3-dependent manner in MuSCs.

### Nrf2 had anti-myogenic and anti-proliferative effects

**Fig 4:**
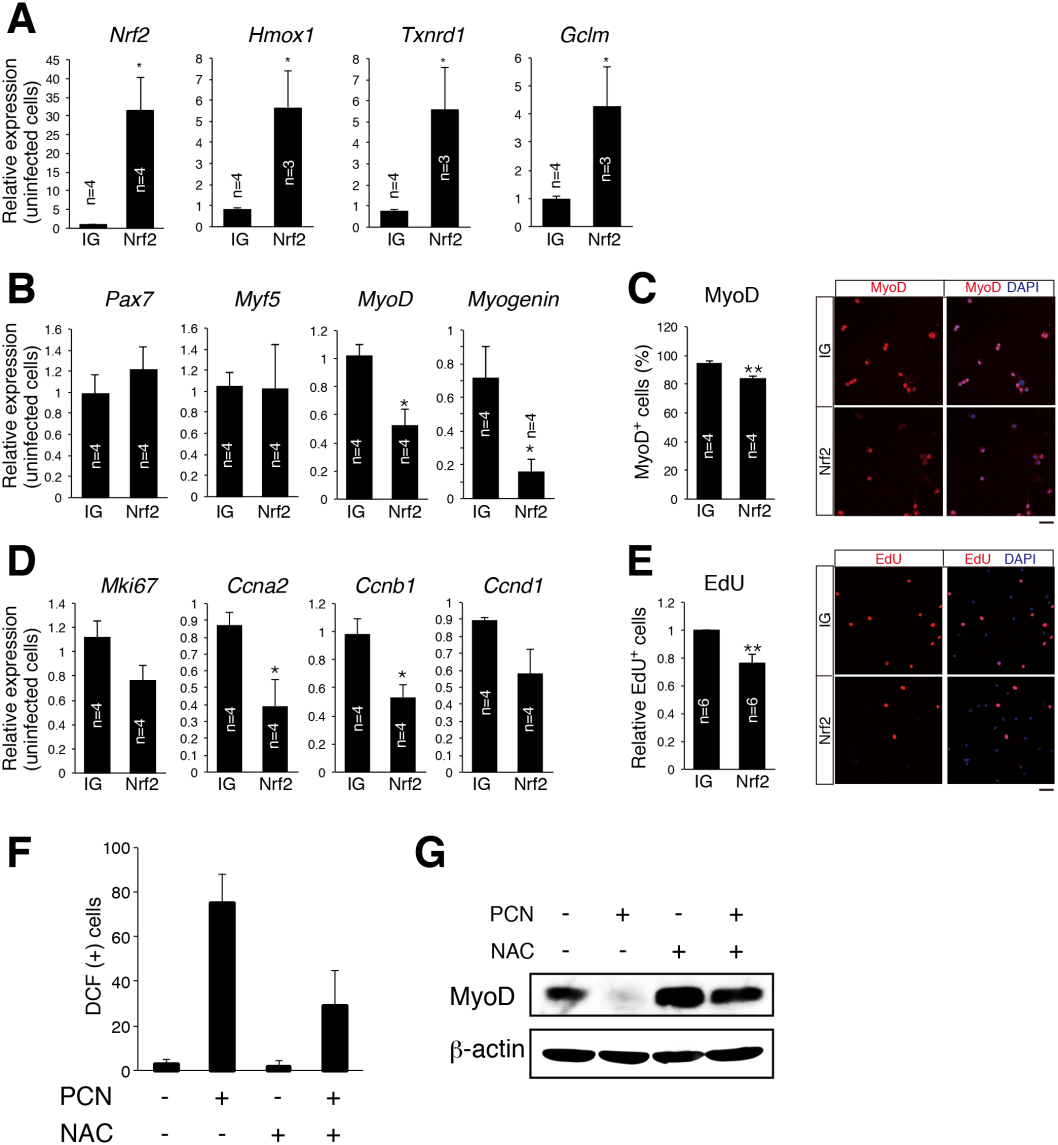
Nrf2 has anti-myogenic and -proliferative effects. (A) Relative expressions of *Nrf2*, *Hmox1*, *Txnrd1*, and *Gclm* mRNA were compared in control (IG), and Nrf2-overexpressed (Nrf2) myoblasts. The *y*-axis indicates means±S.E. (n = 3–4). *, *P*<0.05. (B) Relative expressions of *Pax7*, *Myf5*, *MyoD*, and *Myogenin* mRNA in control (IG), and Nrf2-overexpressed (Nrf2) myoblasts were compared. The *y*-axis indicates means±S.E. (n = 4). *, *P*<0.05. (C) Protein expression of MyoD in control (IG) or Nrf2-overexpressed (Nrf2) myoblasts. The *y*-axis indicates means±S.E. (n = 4). **, *P*<0.01. Scale bar: 50 μm (D) Relative expressions of *Mki67*, *Ccna2*, *Ccnb1*, and *Ccnd1* mRNA were compared in control (IG), and Nrf2-overexpressed (Nrf2) myoblasts. The *y*-axis indicates means±S.E. (n = 4). *, *P*<0.05. (E) The frequency of EdU-positive cells in control (IG) or Nrf2-overexpressed (Nrf2) myoblasts. The *y*-axis indicates means±S.E. (n = 6). **, *P*<0.01. Scale bar: 50 μm (F) Measurement of intracellular ROS level by CM-H_2_DCFDA (DCF) in C2C12 cells cultured with or without PCN or NAC. (G) MyoD protein expressions in C2C12 cells treated with or without PCN or NAC.

In order to examine the impact of Nrf2 on myogenic gene expressions, *Nrf2* genes were retrovirally transduced in primary myoblasts, and mRNA and protein expression of myogenic genes were examined. As shown in Fig 4A, overexpression of *Nrf2* in primary myoblasts resulted in up-regulation of its target anti-oxidative genes, *Hmox1*, *Txnrd1*, and *Gclm* (glutamate-cysteine ligase, modifier subunit) (Fig 4A). In these cells, mRNA expressions of *MyoD* and *myogenin* were inhibited by Nrf2 (Fig 4B). In addition, Nrf2 significantly suppressed MyoD protein in primary myoblasts (Fig 4C).

Next, we examined the effects of Nrf2 on cell proliferation and cell cycle—related gene expressions in primary myoblasts. As shown in Fig 4D, cell cycle—related gene expressions tended to be inhibited by Nrf2. Specifically, *Ccna2* and *Ccnb1* expressions were significantly suppressed by Nrf2. Furthermore, EdU uptake was slightly inhibited by Nrf2 (Fig 4E). Therefore, these results suggested that Nrf2 has anti-myogenic and anti-proliferative effects *in vitro*.

It is well known that the induction of anti-oxidative genes by Nrf2 results in a decreased level of ROS. Therefore, the effect of Nrf2 might result from the reduced level of ROS in primary myoblasts. In order to investigate the correlation between ROS level and myogenesis, C2C12 cells were treated with pyocyanin (PCN), an inducer of oxidative stress. As shown in Fig 4F, PCN increased the ROS level in C2C12 cells, and NAC (N-acetyl-L-cysteine, an inhibitor of ROS) negated the effect of PCN. Using these cells, the MyoD protein level was evaluated by Western blot analyses. As shown in Fig 4G, PCN or NAC significantly inhibited or induced MyoD protein. Therefore, these results suggest that the Nrf2 has an anti-myogenic effect which is likely to stand as a different pathway from the induction of anti-oxidative stress genes by Nrf2.

### *Nrf2*-knockout mice showed no abnormality in skeletal muscle and MuSCs at the basal state

**Fig 5:**
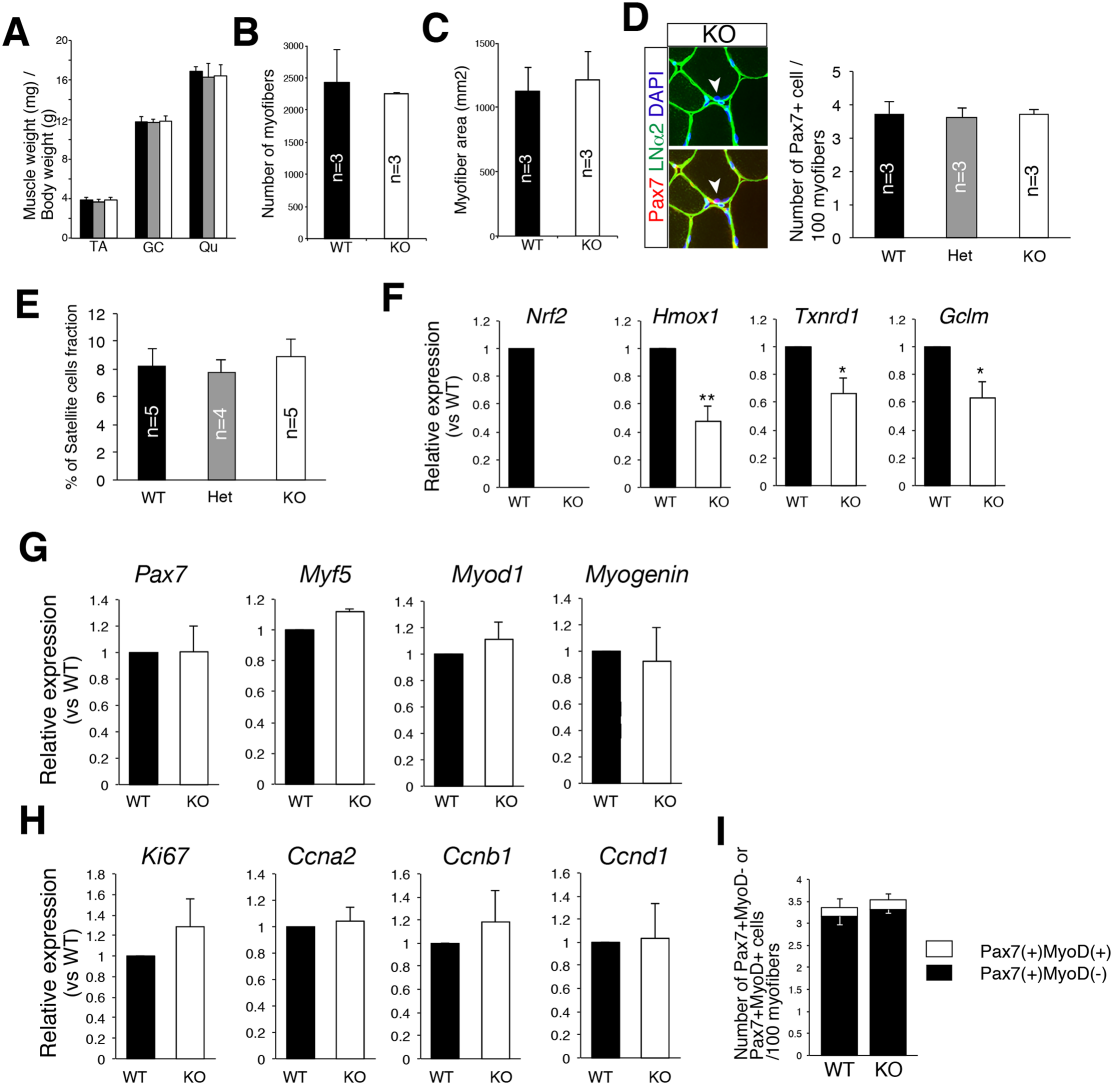
Nrf2-null MuSCs show no sign of abnormality. (A) TA, GC, and Qu muscle weights (mg) per body weight (g) of control (black column), *Nrf2*-heterogyzous (gray column), or *Nrf2*-KO (white column) mice at 15–24 weeks old. The *y*-axis shows the mean with SD. (n = 4–5). (B) The *y*-axis indicates the number of myofibers in control (black column) or *Nrf2*-KO (white column) mice. (n = 3) (C) The *y*-axis indicates the area of myofibers in control (black column) or *Nrf2*-KO (white column) mice. (n = 3) (D) Arrow heads indicate Pax7^+^ (red) cells locating beneath the basal lamina (green; LNα2 laminin α2) in *Nrf2*-KO mice. The *y*-axis indicates the number of Pax7^+^ cells per 100 cross-sectional TA myofibers in control (black column), *Nrf2*-heterogyzous (gray column), or *Nrf2*-KO (white column) mice at 15–24 weeks old. (n = 3) (E) The *y*-axis shows the percentage of the MuSC fraction in control (black column), *Nrf2*-heterogyzous (gray column), or *Nrf2*-KO (white column) mice at 15–24 weeks old. (n = 4–5) (F) Relative expressions of *Nrf2*, *Hmox1*, *Txnrd1*, and *Gclm* mRNA were compared in control (black column) and *Nrf2*-KO (white column) MuSCs. The *y*-axis indicates means±S.E. (n = 4–5). **, *P*<0.01, *, *P*<0.05. (G) Relative expressions of *Pax7*, *Myf5*, *MyoD*, and *Myogenin* mRNA were compared in control (black column) and *Nrf2*-KO (white column) MuSCs. The *y*-axis indicates means±S.E. (n = 3–5). (H) Relative expressions of *Mki67*, *Ccna2*, *Ccnb1*, and *Ccnd1* mRNA of control (black column) and *Nrf2*-KO (white column) MuSCs were compared. The *y*-axis indicates means±S.E. (n = 3). (I) The frequency of Pax7^+^MyoD^-^ or Pax7^+^MyoD^+^ cells. The *y*-axis indicates means±S.E. (n = 3).

In order to determine whether Nrf2 plays physiological roles in the development of skeletal muscle and maintenance of MuSCs, we analyzed *Nrf2*-knockout mice generated previously [14]. As shown in Fig 5A, the muscle weight was not changed among control, *Nrf2*-heterozygous, and *Nrf2*-knockout mice. The number and area of myofibers were also not changed (Fig 5B and 5C). Furthermore, in contrast with hematopoietic stem cells, immunohistochemical studies showed no alternation in the number of MuSC in *Nrf2*-knockout mice (Fig 5D). FACS analyses also supported the lack of effect of *Nrf2*-knockout on MuSC number (Fig 5E). Collectively, *Nrf2*-knockout mice did not show any abnormality in skeletal muscle development or MuSC number under normal condition.

### *Nrf2*-knockout mice showed down-regulation of anti-oxidative genes and normal quiescent and undifferentiated states

We next examined the expressions of myogenic and cell cycle-related genes in *Nrf2*-knockout mice. As shown in Fig 5F, Nrf2 and the target genes of Nrf2 were significantly decreased in *Nrf2*-knockout mice. However, myogenic and cell cycle-related gene expressions of control and *Nrf2*-knockout mice were not different (Fig 5G and 5H). Furthermore, there was no difference in the protein expressions of MyoD between the control and *Nrf2*-knockout group (Fig 5I). We could detect Pax7^+^Ki67^+^ cells, neither in control nor *Nrf2*-knockout mice. Further, the regenerative ability of Nrf2 is similar to the littermate control in cardiotoxin-induced acute damage model (S1 Fig). Collectively, although Nrf2 regulates anti-oxidative genes in MuSCs, Nrf2 is dispensable or has a redundant gene pathway for regulating their quiescent and undifferentiated state.

## Discussion

In this study, we demonstrated that Dll1 induced Nrf2 expression in myogenic cells. In addition, Hesr1 induced Nrf2 expression. Hesr1 is considered to be a transcriptional repressor because it recruits corepressors such as mSin3A, N-CoR, and histone deacetylase 1 via C-terminal YRPW terapeptides [5]. Therefore, the effect of Hesr1 on *Nrf2* expression seems to be indirect. Although Hesr3 has a further degenerated YXXW sequence and the cofactors of Hesr3 are not yet identified, Hesr3 was also considered a transcriptional repressor. *In vitro*, Hesr1 induced the expression of Nrf2, but Hesr3 did not induce it. The decreased level of Nrf2 expression was observed only in *Hesr1*/*Hesr3*-double knockout mice. Hesr1 inhibited MyoD expression, but Hesr3 did not have such an effect in C2C12 cells [15]. A remarkably increased MyoD expression was also observed in only *Hesr1*/*Hesr3*-double knockout mice, demonstrating the complementary roles of hesr1 and hesr3 in satellite cells [8]. Currently, we cannot explain those discrepancies, but one possibility is the co-repressor or co-work molecule of Hesr3 is not expressed in *in vitro* myoblasts for the induction of *Nrf2* mRNA and suppression of MyoD.

In contrast, Rbpj binds directly to the Nrf2 promoter [13]. In our analyses, Dll1 did not successfully induce Nrf2 expression in *Hesr1*/*Hesr3*-double knockout myoblasts. Even in dKO myoblasts co-cultured with control cells, the Nrf2 level was lower than in wild-type cells. These results suggest that the Rbpj-Nrf2 axis is less sensitive or that the Notch-Rbpj-Hesr1/Hesr3-Nrf2 axis is a more crucial pathway in myogenic cells.

There is increasing evidence that Nrf2 and other regulators of the oxidative stress response are critical for quiescence in hematopoietic and neural stem cells. In MuSCs, the loss of *Nrf2* resulted in a decrease in Nrf2 target genes. These results indicate that Nrf2 functions to induce its target genes in MuSCs. However, approximately half of them were still expressed in *Nrf2*-knockout mice, which implies that an Nrf2-independent mechanism also contributes to their expression in MuSCs. Nrf1 is also a member of the Cap’n’collar (Cnc) family of transcriptional factors. During early development, Nrf1 and Nrf2 have overlapping functions and *Nrf1*/*Nrf2*-double knockout fibroblasts showed increased intracellular ROS levels compared with those of each single-knockout mouse [16]. During myogenic development, Pitx2 (Paired-Like Homeodomain 2)/Pitx3 directly regulate Nrf1 expression, and the loss of Pitx2/Pitx3 leads to down-regulation of Nrf1, eventual ROS accumulation, and finally leads to cell death [17]. In MuSCs, *Nrf1* mRNA was detected, and *Nrf2*-knockout mice showed the similar level of *Nrf1* mRNA with the littermate control (S2 Fig). *Nrf2*-overexpressed MuSCs also show same level of *Nrf1* mRNA, indicating Nrf1 might work redundantly with Nrf2 against ROS in MuSCs without influencing Nrf1 expression.

FoxOs (Forkhead box O) genes are also known as transcriptional factors that activate transcription of anti-oxidative genes. In hematopoietic and neural stem cells, the requirement for a FoxO gene or genes is reported [18, 19]. For example, in hematopoietic stem cells, the loss of FoxO3 results in increased cell cycling and reduction of the hematopoietic stem cell pool [20]. Although FoxO3 is expressed in MuSCs and FoxO3 affects their self-renewal, the muscle stem cell pool was not affected one month after FoxO3 deletion as a steady condition [21]. These results might imply that FoxOs and Nrf2 work redundantly in MuSCs.

Although little is known about the function of Nrf2 in MuSCs, the importance of Nrf2 in mouse skeletal muscle was reported. Kombairaju et al. showed that *Nrf2*-knockout A/J mice (a model of dysferlinopathy) exhibited significant muscle-specific functional deficits, histopathologic abnormalities, and dramatically enhanced X-ROS compared to control A/J and WT mice [22]. Others reported that Nrf2 has a minimal role in the mouse skeletal muscle antioxidant defense in two-month-old mice, but Nrf2 disruption promotes oxidative stress and impairs antioxidant mechanisms on aging [23, 24]. Al-Sawaf et al. reported impaired regeneration in *Nrf2*-knockout mice using an ischemia injury model and indicated that MyoD and myogenin promoters contain a highly conserved ARE sequence across species that is the binding site of Nrf2 [25]. In addition, they indicated that Nrf2 induced MyoD or suppressed myogenin expression using 539 bp upstream of the MyoD gene or 4000 bp upstream of the myogenin gene. Intriguingly, the influence of Nrf2 on MyoD and myogenin were detected in proliferating myoblasts, but not in differentiated myotubes. In our analyses, *Nrf2*-knockout mice did not show any defect in skeletal muscle regeneration in acute muscle damaged model (cardiotoxin model), and Nrf2 inhibited MyoD and myogenin expression in proliferating myoblasts. Although further studies are necessary to reveal the expression regulation of myogenic genes by Nrf2, the data provide the evidence that the function of Nrf2 is strongly depended on the state of cell differentiation and injured condition.

ROS accumulation affects myogenic differentiation [26]. For example, *in vitro*, administration of H_2_O_2_ inhibits or slows down the process of myogenic differentiation. In contrast, an ROS trapping agent (phenyl-N-tert-butylnitrone, PBN) reduced redox potentials and enhanced C2C12 differentiation. A similar result was reported by another group [27]. Intriguingly, *p66shc*-knockout mice exhibited lower oxidative stress, and the skeletal muscle of *p66shc*-knockout mice regenerated faster than that of control mice in both ischemia and CTX-injured models. The number of muscle satellite cells in *p66shc*-knockout mice was equal to control mice, but *in vitro* cultured *p66shc*-knockout satellite cells displayed a lower oxidative stress level and a higher proliferation rate and the cells differentiated faster than the control cells. Furthermore, *p66shc*-knockout satellite cells were resistant to H_2_O_2_-induced inhibition of differentiation. It can be suggested that ROS exerts an inhibitory effect on myogenic differentiation. Our results showed the inhibition of MyoD expression by pyocyanin, also, indicating the inhibitory role of ROS on myogenic differentiation. Despite the inhibition of ROS by Nrf2, Nrf2 had a suppressive effect on MyoD expression *in vitro*. Although the anti-oxidative roles of Nrf2 is the best characterized, the inhibitory effect of Nrf2 on MyoD expression seems to be ROS-independent in myogenic cells. In fact, it is reported that Nrf2 plays roles in the survival of hematopoietic stem cells via a ROS-independent mechanism [28].

In summary, we found a potential Notch-Hesr-Nrf2 axis in MuSCs. Although *Nrf2*-knockout mice did not exhibit any impairment in MuSCs, Nrf2 suppresses myogenic and cell-cycle related genes. There is a possibility that MuSCs of aged *Nrf2*-knockout mice show some abnormalities. Because female *Nrf2*-knockout mice spontaneously develop a lupus-like autoimmune nephritis [29], it is difficult to rule out the secondary effect of Nrf2 in MuSCs. Therefore, in order to reveal the direct role of Nrf2 in aged MuSCs, a conditional knockout mouse will be necessary.

## Materials and Methods

### Mice

C57BL/6 mice were purchased from Charles River Japan (Yokohama, Kanagawa, Japan). *Hesr1* ^*-/-*^ mice and *Hesr3* ^*-/-*^ mice were described previously [8, 30]. In order to generate *Hesr1* ^*-/-*^ *Hesr3* ^*-/-*^ mice, *Hesr1* ^*+/-*^ *Hesr3* ^*-/-*^ mice were crossed. *Nrf2* knockout mice [14] were provided by Riken Bio Resource Center (Kanagawa, Japan) and maintained in our animal facility by brother-sister matings. All procedures for experimental animals were approved by the Experimental Animal Care and Use Committee of Osaka University.

### Preparation and FACS analyses of skeletal muscle-derived mononuclear cells

Mononuclear cells from skeletal muscle were prepared using 0.2% collagenase type II (Worthington Biochemical Corp., Lakewood, NJ) as previously described [31]. Myogenic cells were highly purified in the SM/C-2.6^+^CD31^-^CD45^-^Sca-1^-^ fraction [32, 33].

### Immunohistochemistry

Tibialis anterior muscles were isolated and frozen in liquid nitrogen-cooled isopentane (Wako Pure Chemical Industries, Osaka, Japan). For immunohistological analyses, transverse cryosections (6 μm) were fixed with 4% PFA for 10 min. After washing with 0.1% Triton-X100/PBS, endogenous mouse IgG was blocked using an M.O.M. kit (Vector Laboratories Inc., Burlingame, CA), and then reacted with mouse anti-Pax7 and rat anti-laminin α2. The next day, after washing with 0.1% Triton-X100/PBS, the sections were stained with secondary antibodies conjugated with Alexa 488 or Alexa 568 (Molecular Probes, Eugene, OR, USA). The signals were recorded photographically using the confocal laser scanning microscope system TCS-SP5 (Leica, Heerbrugg, Switzerland) or a fluorescence microscope BX51 (Olympus, Tokyo, Japan) equipped with a DP70 CCD camera (Olympus) or BZ-X700 (Keyence, Tokyo, Japan).

### RT-PCR

Total RNA was extracted from sorted cells with a Qiagen RNeasy Micro Kit according to the manufacturer’s instructions (Qiagen, Hilden, Germany) and then reverse-transcribed into cDNA by using TaqMan Reverse Transcription Reagents (Roche Diagnostics, Mannheim, Germany). Real time PCR was performed using SYBR Premix Ex Taq (Takara, Kyoto, Japan) in a final volume of 10 μl. Samples were amplified, and the relative gene expression levels were calculated using standard curves generated by serial dilutions of the cDNA. Specific primer sequences used for PCR are listed in S1 and S2 Tables.

### Retroviral vector preparation and infection experiments

Retrovirus constructs of *Hesr1* and *Hesr3* were described previously [34]. Parental retro CLIG contains GFP, and therefore simultaneously expresses hesr genes and GFP. A full-length *Nrf2* cDNA was amplified by RT-PCR using the following forward or reverse primers containing restrict enzyme sequence, *XhoI* or *NotI*, respectively: forward, 5’-CTCGAGccctcagcatgatggact-3’, and reverse, 5’-GCGGCCGCtcacagtaggaagttttagca-3’. The PCR product was sequenced and cloned into a bicistronic retrovirus construct, *pMXs-IRES/GFP* (a kind gift from T. Kitamura at the University of Tokyo)[35, 36], which contains an IRES-GFP sequence inserted after the *Nrf2* gene. Therefore, it simultaneously expresses Nrf2 and GFP.

The viral particles were prepared as described [35, 36]. After overnight infection with recombinant retroviruses, GFP-positive cells were sorted by FACS Aria II^TM^. To investigate MyoD expression or perform EdU-uptake assays, GFP-positive cells were grown in GM for 2–3 d and then fixed with 4% paraformaldehyde. After permeabilization by 0.25% Triton-X100, the cells were stained with anti-MyoD antibodies (BD Pharmingen, San Diego, CA, USA) or EdU (Invitrogen, Carlsbad, CA, USA). EdU was added to GM 2 h before fixation and was detected following the protocol supplied by the manufacturer. Nuclei were stained with 4,6-diamidino-2-phenylindole (DAPI).

### Satellite cell culture

Freshly isolated satellite cells were cultured in a growth medium (GM) of high-glucose Dulbecco’s modified Eagle’s medium (DMEM-HG; Sigma-Aldrich, St. Louis, MO, USA) containing 20% FCS (Trace Biosciences, N.S.W., Australia), 10 ng/ml bFGF (PeproTech, London, UK), and penicillin (100 U/ml)-streptomycin (100 μg/ml) (Gibco BRL, Gaithersburg, MD) in culture dishes coated with Matrigel (BD Bioscience). Differentiation was induced in differentiation medium (DM) containing DMEM-HG, 5% horse serum, and penicillin-streptomycin for 3–4 d.

### Co-culture with CHO-Dll1

CHO cells stably transfected with mouse Dll1 (CHO-Dll1) or empty vector (CHO-V) were kind gifts from Dr. Zolkiewska (Kansas State University) (Dyczynska et al., 2007). Satellite cells were isolated from wild-type or Hesr1/3-dKO mice, and 1×10^5^ cells were plated in six-well plates. Three days later, CHO-Dll1 or CHO-V were added (5×10^5^ cells/well) and incubated in GM. After 12–24 h, total RNA was extracted from the cultured cells including CHO cells. Primers were designed specific for mouse sequences.

### Measurement of ROS

C2C12 cells were cultured at 2 × 10^5^ cells per well on 6-cm dishes. The next day, pyrocyanin (Sigma-Aldrich) was added at a final concentration of 10 μM. After a 24-h culture, the cells were harvested and analyzed. When the cells were treated with N-acetyl-L-cysteine (NAC), it was added to the cells 1 h before pyrocyanin treatment. ROS was detected by CM-H_2_DCFDA (Life Technologies, Carlsbad, CA, USA) and an FACSCalibur flow cytometer (BD Immunocytometry Systems).

### Western Blot

C2C12 cells were homogenized in lysis buffer containing 50 mM Tris-HCl (pH 7.5), 150 mM NaCl, 1 mM NaF, 1% NP40, and protease inhibitor cocktail (Nacalai Tesque, Kyoto, Japan). The lysate was obtained by centrifugation at 14000 × g for 20 min at 4°C, then the protein concentration was measured by a DC Protein Assay (Bio-Rad Laboratories, Hercules, CA, USA). After addition of 6 × SDS loading buffer (125 mM Tris-HCl, 12% 2-mercaptoethanol, 4% sodium dodecylsulfate, 20% glycerol, and 0.01% bromophenol blue) to the lysate, total proteins were boiled for 5 min and separated by electrophoresis on polyacrylamide gels. Then the proteins were transferred to an Immobilon-P Transfer Membrane (Millipore, Bedford, MA, USA). The membranes were incubated in PBS containing 5% skim milk at room temperature to block non-specific protein binding. The membranes were subsequently incubated in 10 mM Tris-HCl, 150 mM NaCl, and 0.1% Tween 20 buffer containing anti-MyoD antibody or anti-β-actin antibody (Santa Cruz Biotechnology, Santa Cruz, CA, USA) at 4°C overnight. After incubation with the appropriate HRP-labeled secondary antibody (Santa Cruz Biotechnology) for 1 h at room temperature, the blots were stained with an ECL Western blotting kit (GE Healthcare, Little Chalfont, UK). The signals were detected using a Light-Capture imaging system (Atto Corp., Tokyo, Japan).

### Statistics

Values were expressed as means ± SD or SE. Statistical significance was assessed by Student’s *t* test. In comparisons of more than two groups, non-repeated measures analysis of variance (ANOVA) followed by the Bonferroni test (vs. control) or SNK test (multiple comparisons) were used. A probability of less than 5% (*p*<0.05) or 1% (*p*<0.01) was considered statistically significant.

## Supporting Information

S1 Fig The ability of skeletal muscle regeneration in Nrf2-KO mice. TA muscles of Nrf2-KO and the littermate control mice were damaged by cardiotoxin. After 2 weeks, the TA muscles were fixed and stained by H&E.
(PDF)

S2 Fig Expression level of Nrf1 mRNA in Nrf2-KO MuSCs or Nrf2-overexpressing myoblasts. (A) Relative expressions of Nrf1 mRNA in wild type (WT) and Nrf2-KO (KO) MuSCs. The y-axis indicates means±S.E. (n = 5). (B) Relative expressions of Nrf1 mRNA control (IG), and Nrf2-overexpressed (Nrf2) myoblasts were compared. The y-axis indicates means±S.E. (n = 4).
(PDF)

S1 Table Sequences of primers for standard. Primer sequences and product size are listed.
(DOCX)

S2 Table Sequences of primers for real-time PCR. Primer sequences and product size are listed.
(DOCX)
