# Supplementary figures and images for: Evidence of Notch-Hesr-Nrf2 Axis in Muscle Stem Cells, but Absence of Nrf2 Has No Effect on Their Quiescent and Undifferentiated State
Source: PLoS One. 2015 Sep 29;10(9):e0138517. doi: 10.1371/journal.pone.0138517 (PMC4587955; doi:10.1371/journal.pone.0138517)

Supplemental Figure 1

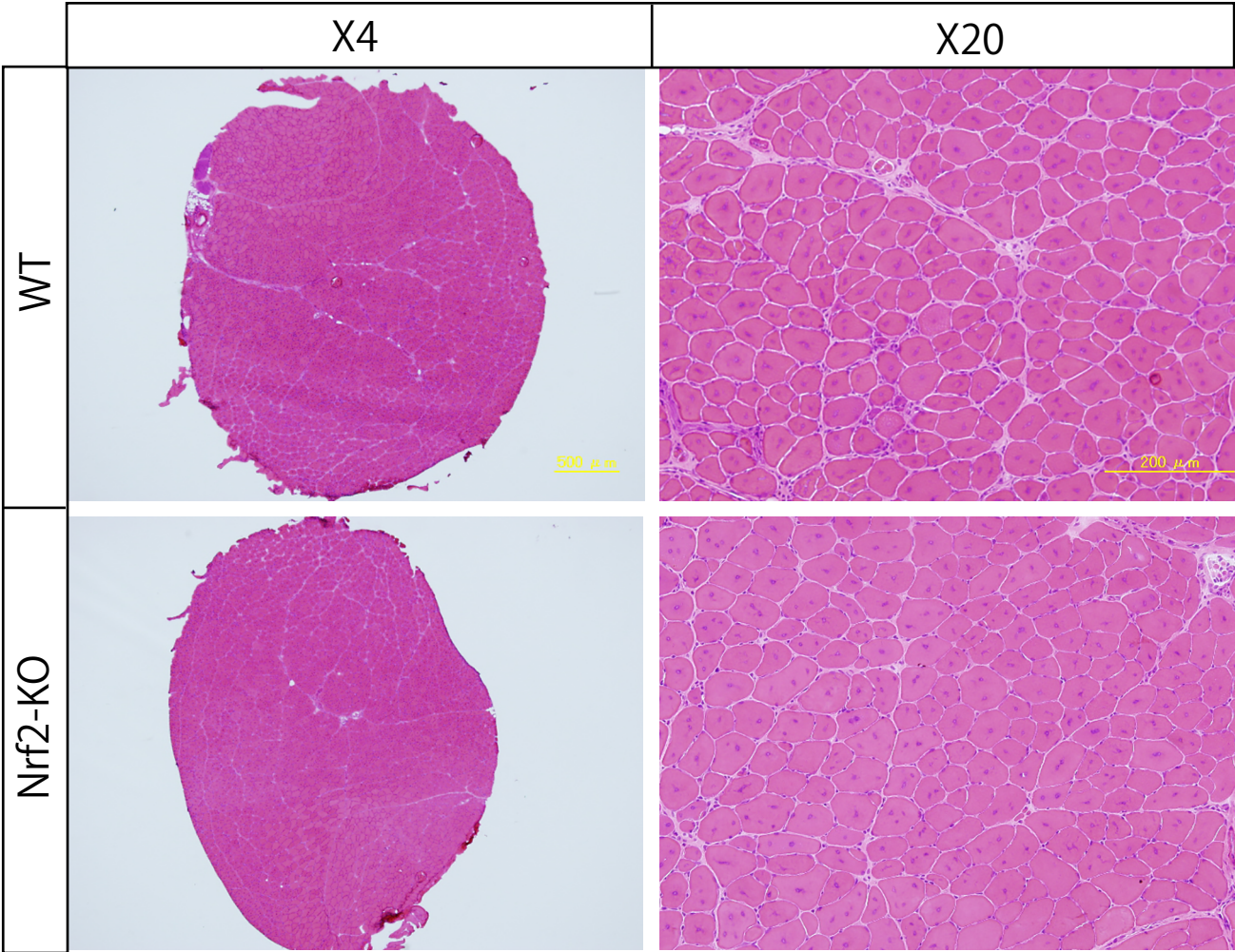

Supplement: S1 Fig — TA muscles of Nrf2-KO and the littermate control mice were damaged by cardiotoxin. After 2 weeks, the TA muscles were fixed and stained by H&E. (PDF) [file pone.0138517.s001.pdf]

Supplemental Figure 2

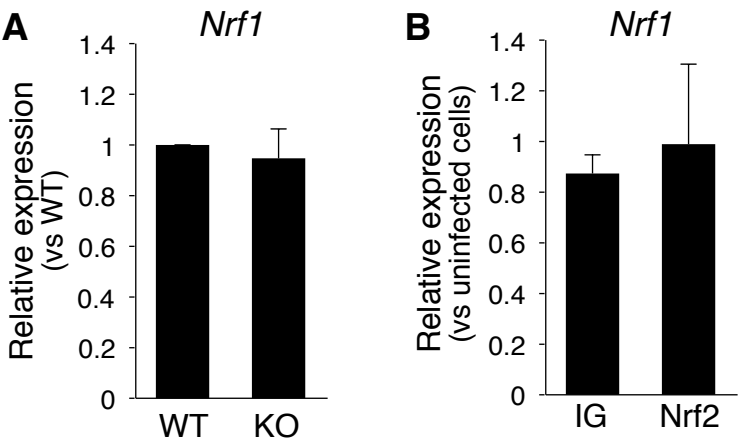

Supplement: S2 Fig — (A) Relative expressions of Nrf1 mRNA in wild type (WT) and Nrf2-KO (KO) MuSCs. The y-axis indicates means±S.E. (n = 5). (B) Relative expressions of Nrf1 mRNA control (IG), and Nrf2-overexpressed (Nrf2) myoblasts were compared. The y-axis indicates means±S.E. (n = 4). (PDF) [file pone.0138517.s002.pdf]

**Table S1: Sequences of primers for standard**
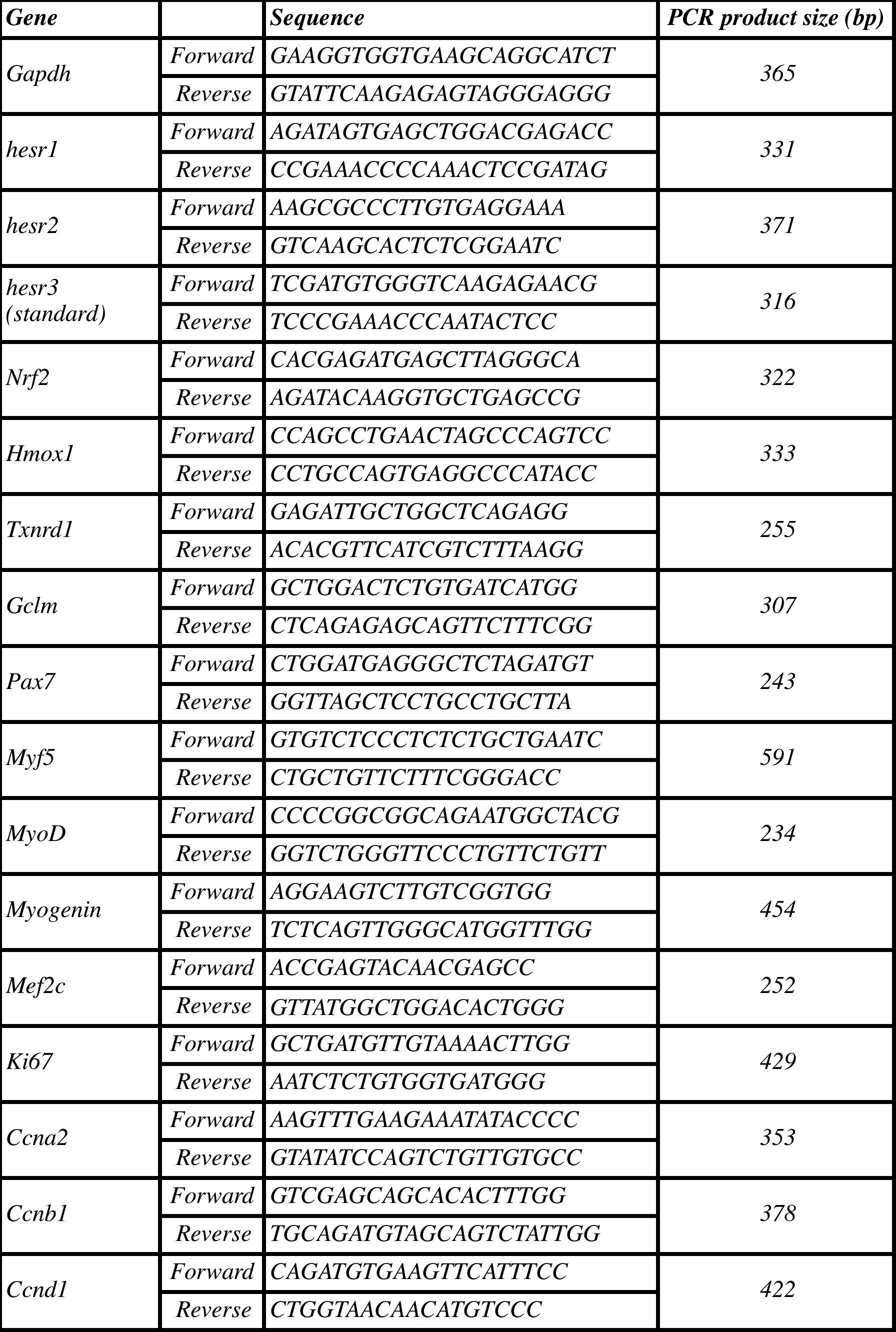

Supplement: S1 Table — Primer sequences and product size are listed. (DOCX) [file pone.0138517.s003.docx]

**Table S2: Sequences of primers for real-time PCR**


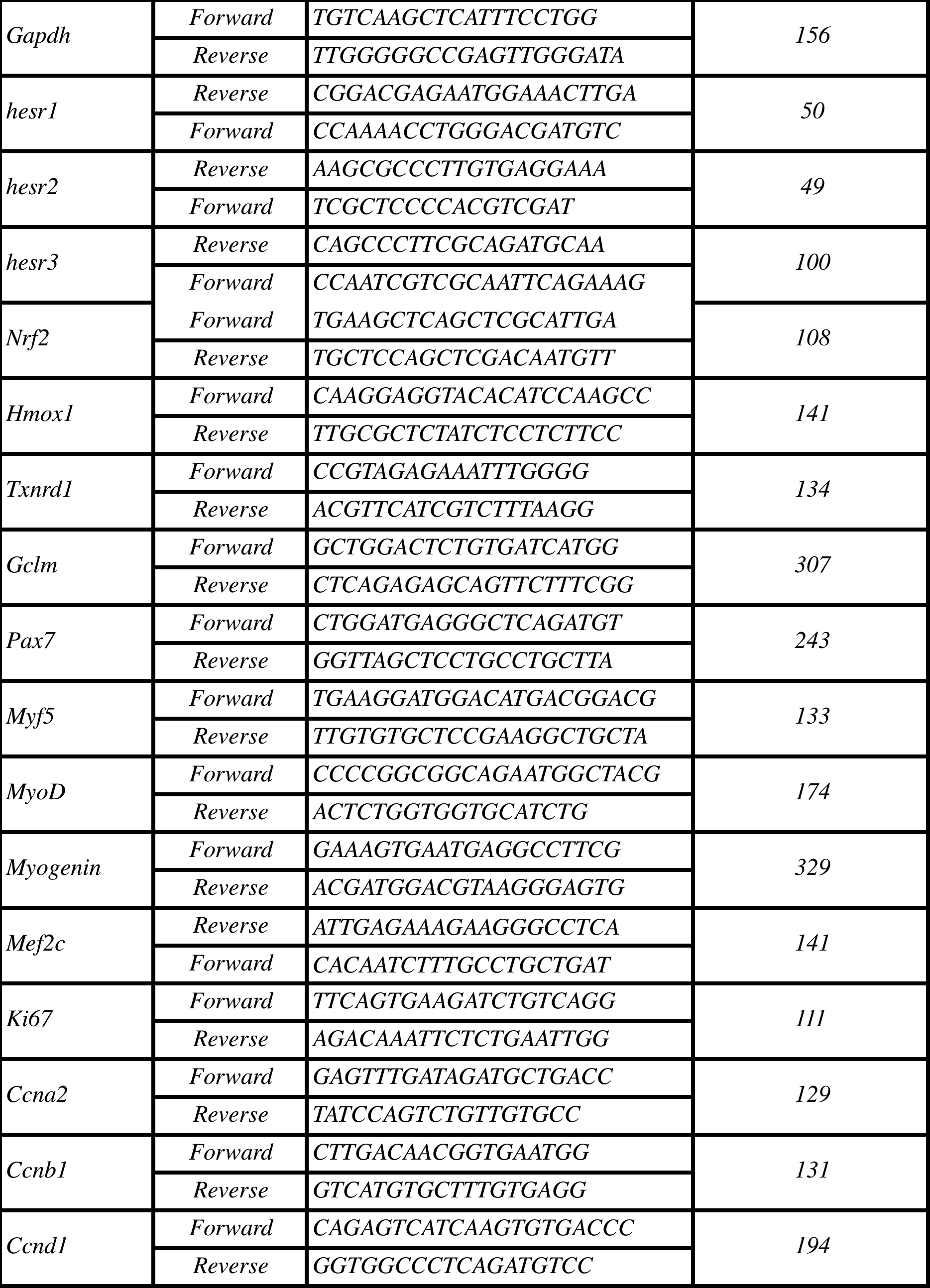

Supplement: S2 Table — Primer sequences and product size are listed. (DOCX) [file pone.0138517.s004.docx]
